# Role of Adiponectin Receptor 1 in Promoting Nitric Oxide-Mediated Flow-Induced Dilation in the Human Microvasculature

**DOI:** 10.3389/fphar.2022.875900

**Published:** 2022-04-04

**Authors:** Katie E. Cohen, Boran Katunaric, Mary E. Schulz, Gopika SenthilKumar, Micaela S. Young, James E. Mace, Julie K. Freed

**Affiliations:** ^1^ Department of Medicine-Division of Cardiovascular Medicine, Medical College of Wisconsin, Milwaukee, WI, United States; ^2^ Cardiovascular Center, Medical College of Wisconsin, Milwaukee, WI, United States; ^3^ Department of Anesthesiology, Medical College of Wisconsin, Milwaukee, WI, United States; ^4^ Department of Physiology, Medical College of Wisconsin, Milwaukee, WI, United States; ^5^ Department of Surgery-Division of Adult Cardiothoracic Surgery, Medical College of Wisconsin, Milwaukee, WI, United States

**Keywords:** adiponectin, nitric oxide, microvascular, endothelium, flow, dilation

## Abstract

Chronic administration of exogenous adiponectin restores nitric oxide (NO) as the mediator of flow-induced dilation (FID) in arterioles collected from patients with coronary artery disease (CAD). Here we hypothesize that this effect as well as NO signaling during flow during health relies on activation of Adiponectin Receptor 1 (AdipoR1). We further posit that osmotin, a plant-derived protein and AdipoR1 activator, is capable of eliciting similar effects as adiponectin. Human arterioles (80–200 μm) collected from discarded surgical adipose specimens were cannulated, pressurized, and pre-constricted with endothelin-1 (ET-1). Changes in vessel internal diameters were measured during flow using videomicroscopy. Immunofluorescence was utilized to compare expression of AdipoR1 during both health and disease. Administration of exogenous adiponectin failed to restore NO-mediated FID in CAD arterioles treated with siRNA against AdipoR1 (siAdipoR1), compared to vessels treated with negative control siRNA. Osmotin treatment of arterioles from patients with CAD resulted in a partial restoration of NO as the mediator of FID, which was inhibited in arterioles with decreased expression of AdipoR1. Together these data highlight the critical role of AdipoR1 in adiponectin-induced NO signaling during shear. Further, osmotin may serve as a potential therapy to prevent microvascular endothelial dysfunction as well as restore endothelial homeostasis in patients with cardiovascular disease.

## Introduction

The traditional view of the microvasculature as a network of small resistance arterioles that regulate end-organ perfusion has expanded to being a critical modulator of underlying parenchyma which can prevent or promote cardiovascular disease. Coronary, as well as peripheral systemic microvascular dysfunction, are powerful predictors of future major adverse cardiac events (MACE) (TPvd et al., 2014). Microvascular endothelial dysfunction, or a state of reduced nitric oxide (NO) bioavailability due to increased oxidative stress, can be assessed by determining the endothelial vasoactive mediator formed during flow that results in dilation (flow-induced dilation; FID). Under physiological conditions, the FID mediator generated is NO, an anti-inflammatory, anti-thrombotic compound whereas arterioles from patients with CAD rely on the formation of the pro-inflammatory, pro-atherosclerotic compound H_2_O_2_ to elicit dilation.

We have previously shown that chronic exposure (16–20 h) to adiponectin, or a nonselective adiponectin receptor agonist (AdipoRON), is capable of restoring NO-mediated FID in human microvessels collected from patients diagnosed with CAD (Schulz et al., 2019). Adiponectin is a unique adipokine with many reported beneficial effects on the vasculature. These vascular effects include but are not limited to, triggering NO formation through activation of endothelial nitric oxide (Hattori et al., 2003) (eNOS) and promoting breakdown of ceramide (Holland et al., 2011), a sphingolipid that when elevated in plasma predicts future MACE and induces microvascular endothelial dysfunction (Freed et al., 2014a; Akhiyat et al., 2021). Targeting the adiponectin pathway may offer a therapeutic strategy to prevent or reverse microvascular endothelial dysfunction before the formation of large artery disease. Here we hypothesized that activation of adiponectin receptor 1 (AdipoR1) is responsible for the restoration of NO-mediated FID in microvessels from subjects with disease. The question was addressed here by examining 1) the expression of AdipoR1 in microvessels from patients with and without CAD, 2) the role of AdipoR1 in adiponectin-induced restoration of NO as the primary mediator of FID in diseased arterioles, and 3) whether osmotin, a plant-derived stress protein whose receptor shares homology with AdipoR1, can serve in the same capacity and promote vasodilation that relies on the formation of NO. The translational studies reported here highlight the importance of AdipoR1 signaling and offer mechanistic insight into the role of adiponectin, and potentially osmotin, in promoting a quiescent human microvascular endothelium.

## Materials and Methods

### Human Tissue Acquisition

Fresh human adipose deemed discarded was collected at the time of surgery and placed in ice-cold HEPES buffer (pH 7.4). Discarded tissue was transported to the Pathology department (honest broker) housed within the hospital, then sent to the laboratory for dissection. De-identified patient data is collected using RedCap, a clinical research database at the Medical College of Wisconsin. In addition to the de-identified discard collection, clinical research coordinators are approved to screen for operations that will likely result in discard tissue from the procedure and consent patients to collect their surgical discard tissue along with more detailed patient information. All protocols were approved by the local Institutional Review Board (IRB) at the Medical College of Wisconsin.

### Videomicroscopy for Microvascular Function Studies

The microvascular functional study technique has been extensively used by our laboratory (Miura et al., 2003). In an organ chamber, both ends of the resistance vessel (80–200 μm) are cannulated with glass micropipettes filled with physiological saline solution and pressurized (60 mmHg) prior to measuring internal diameter change using videomicroscopy. Administration of endothelin-1 (2 nM) was used to pre-constrict the vessel to 30–70% of its passive diameter prior to initiating flow. Flow-induced dilation (FID) was measured as originally described by Kuo, et al. (Kuo et al., 1990) using pipettes of matched impedance. Changing the height of each reservoir in equal amounts in opposite directions generated flow without changing vessel central pressure (Kuo et al., 1990). Data are reported as diameter at a given flow rate or pressure gradient.

Internal diameters were assessed 5 min (steady-state) after each change. Two flow-response curves were generated, one after adding vehicle, and one after inhibitors (e.g. L-NAME). The concentration of agents was determined by multiplying the ED_50_ 2 × for inhibition or activation for each specific compound. Based on our previous studies, 16–20 h is an adequate duration of incubation for assessing the chronic effect of pharmacological agents. Acute treatment is defined as 30 min - 4 h of drug exposure either in the chamber bath, or in a dish containing culture media. L-NAME (100 μM) or cPTIO (100 μM) were added to the chamber bath to determine the role of NO in FID whereas the addition of PEG-catalase (500 U) was used to assess the contribution of H_2_O_2_ to dilation. Smooth muscle function was assessed at the completion of each flow experiment by measuring the dilator response to papaverine (100 μM).

### siRNA Transfection

Knockdown of AdipoR1 was achieved as previously described (Kadlec et al., 2017). Silencer Select Negative Control siRNA was used as a control. Briefly, arterioles were cannulated and transfused with the negative control or target protein siRNA (50 nM) with Lipofectamine RNAiMAX (Invitrogen). The ends of each arteriole were tied off to allow for intraluminal exposure and endothelial targeted knockdown of AdipoR1. Vessels were then incubated for 4 h in fresh medium. The following day, ties were cut off the ends of the arterioles prior to cannulation for functional studies. Knockdown efficiency was evaluated using immunofluorescence.

### Immunofluorescence

Dissected resistance arterioles from human adipose tissue were fixed in 10% neutral buffer formalin for up to 72 h. After fixation, tissues were dehydrated through graded ethanol, cleared with xylene, and paraffin infiltrated using automated tissue processing (Sakura Tissue TEK VIP5 and VIP6). Tissue cassettes are embedded into paraffin blocks following processing. Tissue blocks were sectioned at 4 μm and mounted on poly-L-lysine coated slides until subsequent staining. Staining was performed on a Leica Bond RX automated staining platform. Deparaffinization and citrate buffer retrieval was performed prior to staining protocol. After protein blocking (30 min, DAKO X0909) antibodies for AdipoR1 and CD31 were combined as a cocktail and incubated for 1 h at room temperature. After TBST washing, Donkey anti Mouse-488 (Invitrogen A10037) was cocktailed with Donkey anti Rabbit Cy3(Jackson Immuno 715-166-152) and incubated 45 min at room temperature. Nuclei were stained with DAPI (Sigma, D8417). Omission of primary antibody performed for negative reagent controls. Fluorescent images of the vessel cross sections were acquired on an Olympus inverted fluorescent microscope. Image analysis was completed in Image J by outlining the outer and inner layer of the endothelium (marked by CD31) and applying the outline to quantify endothelium specific AdipoR1. The mean gray value of the endothelial lining is reported as mean ± SEM.

## Materials

Adiponectin (Recombinant Human gAcrp30/Adipolean, PeproTech US, Rocky Hill, NJ, United States) was reconstituted in 0.1% 1% bovine serum albumin (BSA). Endothelin-1 (MilliporeSigma, St. Louis, MO, United States) was dissolved in 1% BSA solution. Nω‐nitro‐L‐arginine methyl ester (L‐NAME) and polyethylene glycol-catalase (PEG-catalase) (Millipore Sigma) and 2-(4-carboxyphenyl)-4,4,5,5-tetramethylimidazoline-1-oxyl-3-oxide (cPTIO) (Cayman Chemical, Ann Arbor, MI, United States) were dissolved in water. Osmotin (Abcam, Cambridge, United Kingdom) was diluted in a Tris/glycine buffer at pH 7.4. Anti-AdipoR1 antibody (ab126611) was also obtained from Abcam. Anti-AdipoR1 siRNA (s27410) and negative control siRNA (Silencer Select Negative Control #1) were obtained from Invitrogen by Thermo Fisher (Thermo Fisher Scientific, Waltham, MA, United States).

### Statistical Analysis

All data presented are expressed as mean ± SEM. Percent maximal dilation is calculated as a percentage of maximal relaxation following constriction with endothelin-1. 100% dilation is full relaxation to the maximum diameter observed from the addition of the smooth muscle relaxant papaverine at the end of the flow experiment. A 2-way ANOVA was used with treatment and flow gradient used as parameters. The Holm-Sidak multiple comparisons test was used when significant differences (*p* < 0.05) were observed between treatments. Statistical analysis was performed using GraphPad Prism, version 9.3.0. Statistical significance was defined as *p* < 0.05.

## Results

Discarded surgical adipose was collected from a total of 36 patients for this study. The majority of microvessels were dissected from adipose collected from patients formally diagnosed with CAD (30) with the remaining (6) collected from nonCAD patients who had 0–1 risk factor for CAD (hypertension, hyperlipidemia, diabetes mellitus, congestive heart failure, active smoker). Demographic data including age, sex, BMI, race, and underlying disease or risk factors is presented in Table 1.

**TABLE 1:** Patient Demographics. CAD, coronary artery disease; BMI, body mass index; M, male; F, female. n indicates number of patients.

Characteristics	NonCAD (*n* = 6)	CAD (*n* = 30)
Sex, M/F	6	21/9
Age, years (average ± SD)	44 ± 12	66 ± 8
BMI(average ± SD)	31 ± 4	32 ± 5
Race	Caucasian (*n* = 5)	Caucasian (*n* = 26)
	Hispanic (*n* = 1)	African American (*n* = 5)
	—	Asian/Pacific Islander (*n* = 1)
	—	Unknown (*n* = 1)
Underlying diseases/risk factors		
Coronary artery disease	0	30
Hypertension	1	22
Hyperlipidemia	1	22
Diabetes mellitus	0	9
Active smoker	0	5
Congestive heart failure	0	7
None of the above	4	0

### Expression of AdipoR1 in the Human Microcirculation

**FIGURE 1:**
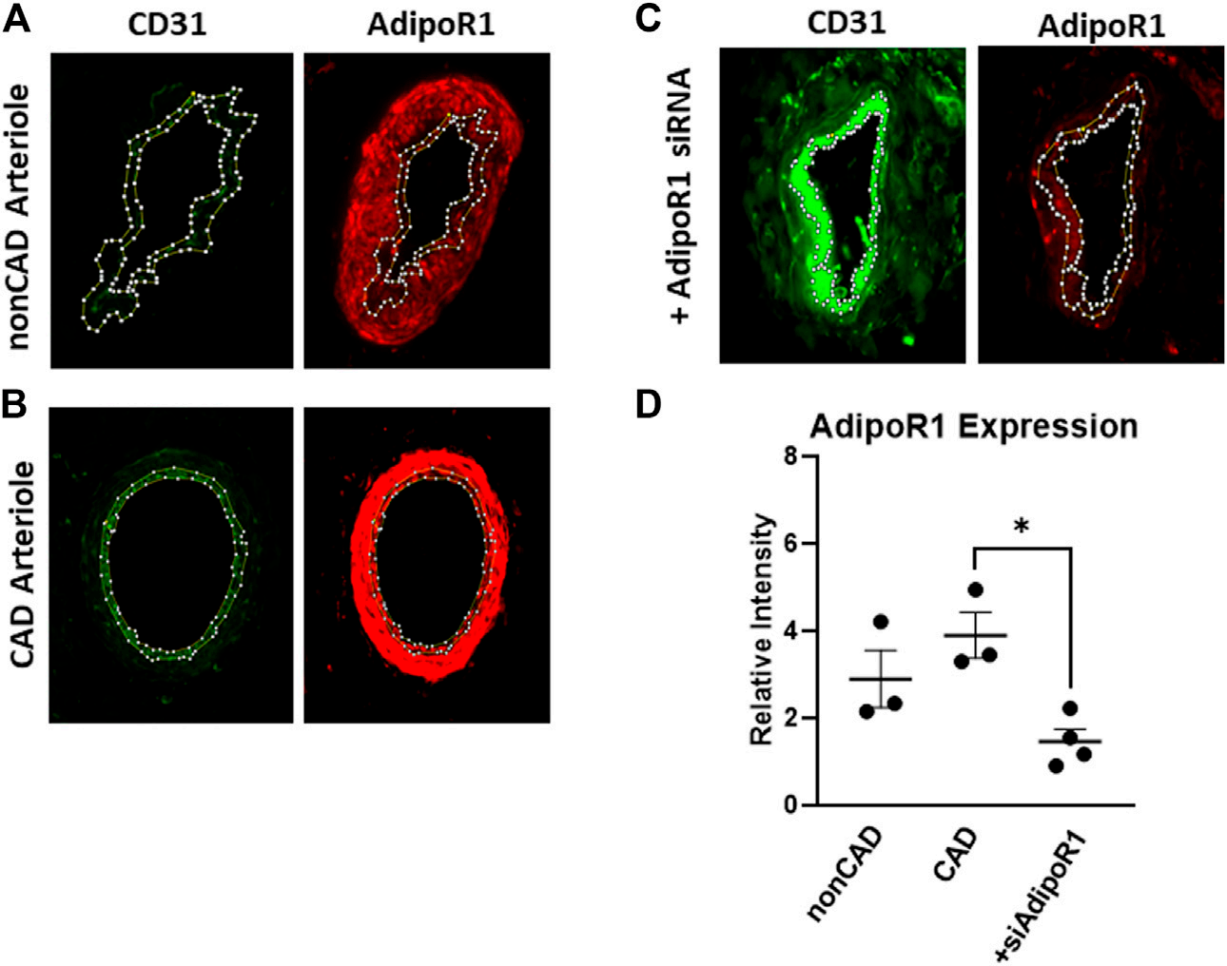
AdipoR1 expression in human microvessels. Representative images showing expression of CD31 (green) and AdipoR1 (red) in arterioles from patients without CAD **(A)**, with CAD **(B)** and arterioles treated with siAdipoR1 **(C)**. CD31 was used to identify the endothelium to allow for measurement of AdipoR1 in the intimal layer of the vessel. **(D)** Measured relative intensity of AdipoR1 in all groups. (*n* = 3 for nonCAD and CAD, *n* = 4 for vessels treated with siAdipoR1; 3 nonCAD, 1 CAD), *p < 0.05).

Resistance arterioles were dissected from human adipose tissue collected from both nonCAD and CAD patients for detection of AdipoR1. Figure 1 shows representative images of human arterioles treated with immunofluorescent antibodies against AdipoR1. AdipoR1 expression in arterioles from nonCAD patients is shown in Figure 1A and patients diagnosed with CAD in Figure 1B. Microvessels from individuals with and without CAD were treated intraluminally with siRNA against AdipoR1 Figure 1C. Cross-sections of the arterioles were immunolabeled for CD31 to outline the endothelium and allow for identification of endothelial AdipoR1. Figure 1D summarizes the immunofluorescent intensity in all 3 groups. AdipoR1 is expressed in human microvessels during both health and disease and expression is significantly decreased in arterioles treated with siAdipoR1.

### Decreased Expression of AdipoR1 Impairs Restoration of Nitric Oxide-Mediated Flow-Induced Dilation

**FIGURE 2:**
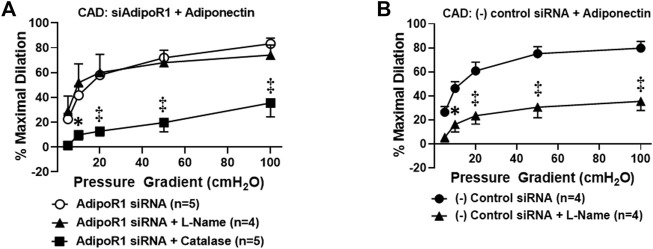
Adiponectin restores NO-dependent FID *via* activation of AdipoR1 in CAD microvessels. **(A)** FID is significantly decreased in arterioles collected from patients with CAD treated with both siAdipoR1 and adiponectin in the presence of PEG-catalase (*n* = 5) compared to vessels treated with siAdipoR1 alone (*n* = 5) whereas L-Name had no effect (*n* = 4). **(B)** Intraluminal treatment with negative control siRNA does not impair adiponectin-induced restoration of NO as the mediator of FID as dilation is inhibited in the presence of L-NAME (*n* = 4) compared to negative control siRNA alone (*n* = 5). ^‡^ *p* < 0.01, *p < 0.05 versus AdipoR1 siRNA alone. n indicates the number of patients.

We have previously shown that chronic exposure to exogenous adiponectin restores NO as the mediator of FID in arterioles collected from patients with CAD (Schulz et al., 2019). To determine whether AdipoR1 is responsible for this effect, microvessels from patients with CAD were first treated with siRNA against AdipoR1 for 4 h prior to incubation with exogenous adiponectin for 16–20 h. As shown in Figure 2A, FID is significantly impaired in vessels treated with both siAdipoR1 and adiponectin in the presence of PEG-catalase [%maximal dilation (MD) 35.5 ± 11.1, *n* = 4, ^‡^ *p* < 0.01 versus 83.2 ± 4.6, *n* = 5; siAdipoR1 alone] whereas L-NAME had no effect (%MD 74.1 ± 8.1, *n* = 4), suggesting that the primary mediator remains H_2_O_2_ despite treatment with adiponectin. Restoration of NO-mediated FID was achieved in arterioles from patients with disease treated with a negative control siRNA (4 h) prior to adiponectin (16–20 h) as evidenced by significant decreased dilation during exposure to L-NAME (%MD 35.4 ± 7.6, *n* = 4 versus 80.0 ± 4.3, *n* = 5; negative control siRNA alone, Figure 2B).

### Role of AdipoR1 in Flow-Induced Dilation During Health and Disease

**FIGURE 3:**
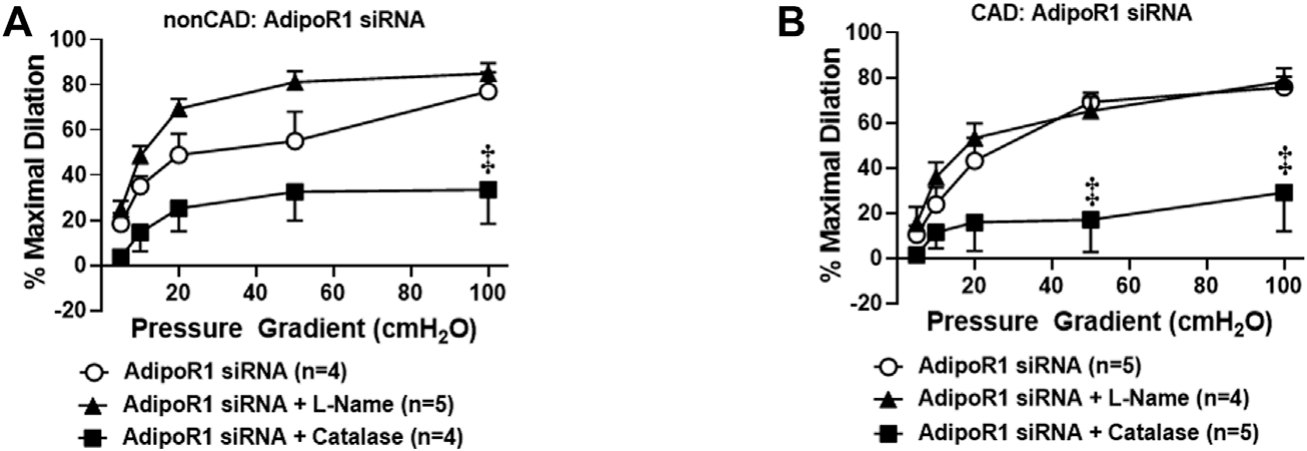
Role of AdipoR1 in FID within the human microvasculature. **(A)** FID in nonCAD arterioles with reduced expression of AdipoR1 was significantly impaired in the presence of PEG-catalase (*n* = 4) compared to siAdipoR1 alone (*n* = 4, ^‡^ *p* < 0.01) whereas L-NAME had no effect (*n* = 5). **(B)** Dilation to flow in arterioles from CAD subjects treated with siAdipoR1 and PEG-Catalase (*n* = 5) was significantly decreased compared to vessels treated with siAdipoR1 alone (*n* = 6, ^‡^ *p* < 0.01), however L-Name had no effect (*n* = 4).

Prior studies have shown that adiponectin receptors have the intrinsic ability to hydrolyze ceramide to sphingosine (Vasiliauskaité-Brooks et al., 2017) and thus can prevent accumulation of intracellular ceramide. We have previously shown that exogenous ceramide is capable of triggering the transition in FID mediator from NO to H_2_O_2_ in arterioles from healthy, nonCAD adults, the same change in mechanism that occurs between health and disease (Freed et al., 2014b). To understand the role of adipoR1 in the mediator formed due to flow during health, AdipoR1 expression was decreased in arterioles from nonCAD adults prior to functional analysis. Figure 3A illustrates that following knock-down of AdipoR1, FID is impaired in arterioles exposed to PEG-catalase (%MD 33.4 ± 15.0, *n* = 4) compared to siAdipoR1 alone (%MD 77.1 ± 8.4, *n* = 4, ^‡^ *p* < 0.01). Treatment with L-NAME had no effect on maximal dilation in response to flow (%MD 84.9 ± 4.6, *n* = 5) confirming that decreased expression of AdipoR1 alone may promote H_2_O_2_-dependent FID.

The mechanism of FID was also examined in arterioles from CAD patients treated with siAdipoR1 as shown in Figure 3B. Following treatment with siAdipoR1, FID remained dependent on H_2_O_2_ as vasodilatory capacity was significantly decreased during exposure to PEG-catalase (%MD 29.0 ± 17.2, *n* = 5; PEG-catalase versus 75.8 ± 4.6, *n* = 5; siAdipoR1 alone) whereas L-NAME had no effect (%MD 78.3 ± 6.0, *n* = 4).

### Osmotin as a Potential Activator of AdipoR1 in the Human Microvasculature

**FIGURE 4:**
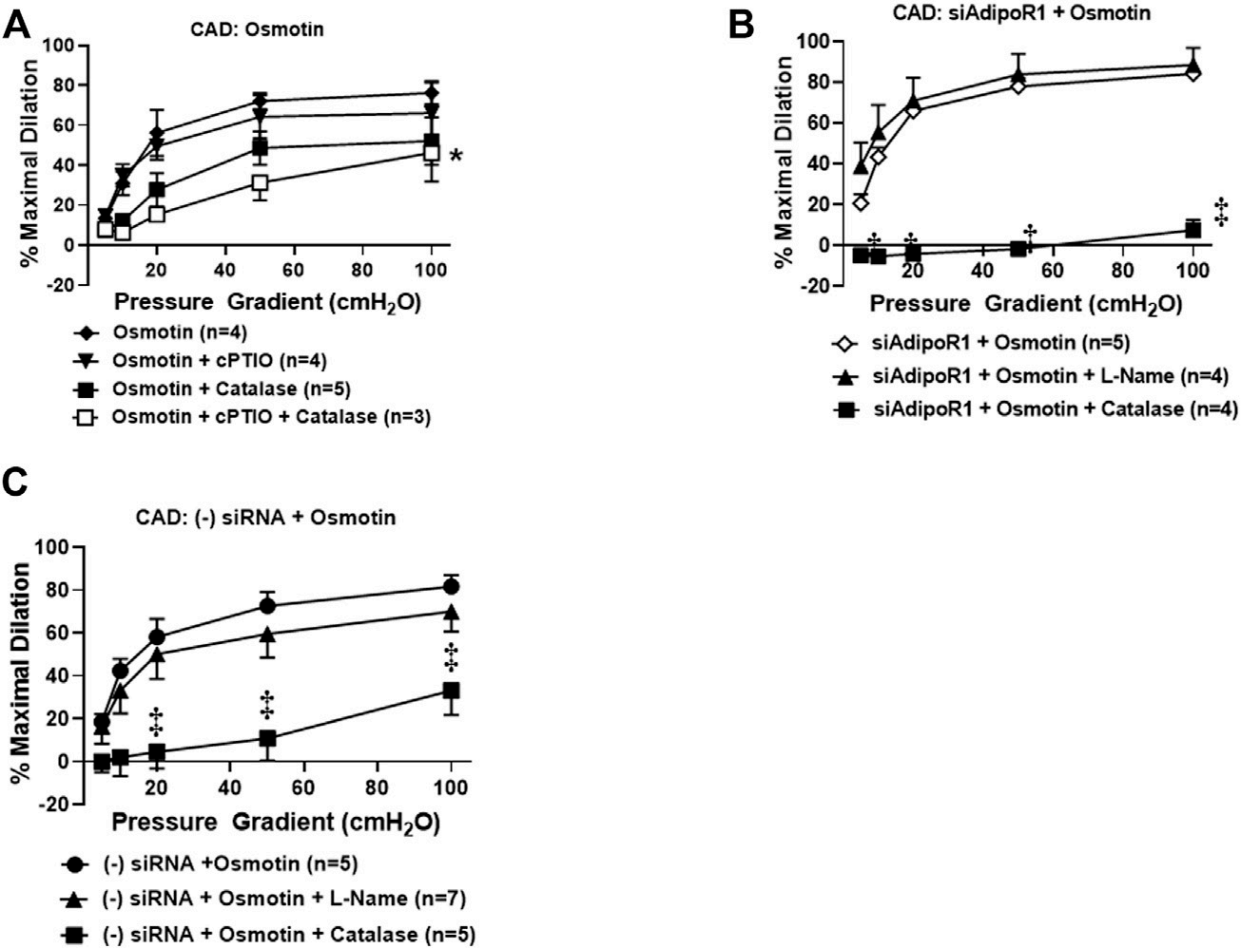
Osmotin as potential activator of AdipoR1. **(A)** FID in CAD arterioles treated with osmotin (0.3 μM, 16–20 h) was not reduced in the presence of the NO scavenger cPTIO (*n* = 4) or PEG-catalase (*n* = 5). Significant reduction of maximal dilation was only observed with both inhibitors (*n* = 3, *p < 0.05). **(B)** The effect of osmotin was eliminated in arterioles from CAD patients with reduced expression of AdipoR1 as PEG-catalase significantly decreased overall dilation (*n* = 4) compared to siAdipoR1 and osmotin (*n* = 5, ^‡^ *p* < 0.01) whereas L-Name had no effect (*n* = 4). **(C)** The primary mediator of FID remains dependent on H_2_O_2_ despite osmotin treatment in arterioles treated with negative control siRNA (*n* = 5 for both PEG-catalase treated and no inhibitor, ^‡^ *p* < 0.01) as opposed to NO (*n* = 7; L-Name).

Our prior work has suggested that restoration of NO-mediated FID in arterioles from CAD patients is also achieved using AdipoRON (Schulz et al., 2019), a nonselective activator of AdipoR1 and R2 (Liu et al., 2019; Salvator et al., 2021). Osmotin is a plant-derived protein that has proven crucial to tolerate stress in most fruits and vegetables (Qureshi et al., 2007) and exhibits structural and functional homology with adiponectin (Min et al., 2004). This stress protein activates PHO36, a receptor found in plants and whose homolog in humans is AdipoR1 (Narasimhan et al., 2005). We therefore hypothesized that exogenous treatment with osmotin would restore NO-mediated FID in arterioles from diseased patients, similar to what is observed with exogenous adiponectin. To test this, arterioles from patients with CAD were incubated with osmotin (0.3 μM, 16–20 h) prior to functional studies to determine the mediator produced during flow. Here, arterioles from subjects with CAD dilated to flow in the presence of the NO scavenger, cPTIO (%MD 66.1 ± 15.2, *n* = 4) or PEG-catalase (%MD 52.2 ± 11.8, *n* = 5) compared to osmotin with no inhibitors (%MD 76.2 ± 5.9, *n* = 6). Although maximal dilation was reduced, FID was only significantly impaired when both agents were added simultaneously to the bath (%MD 46.3 ± 14.3, *n* = 3, Figure 4A), suggesting that both of these mediators may be contributing to dilation from flow. The dual mediator phenotype is abolished in CAD vessels treated with both siAdipoR1 and osmotin as L-NAME had no effect on dilation (%MD 88.7 ± 8.4, *n* = 4, Figure 4B) whereas PEG-catalase significantly reduced vasodilatory capacity compared to vessels with no inhibitor present (%MD 7.6 ± 5.2, *n* = 4 versus 84.3 ± 2.8, *n* = 5, respectively). Interestingly, arterioles from diseased patients treated with negative control siRNA and osmotin did not display the dual mediator phenotype (Figure 4C). FID remained dependent on H_2_O_2_ as only the presence of PEG-catalase significantly reduced maximal dilation compared to vessels not treated with inhibitors (%MD 33.2 ± 11.5, *n* = 5 versus 81.7 ± 5.3, *n* = 5, respectively) whereas L-NAME had no effect (%MD 70.0 ± 9.3, *n* = 7).

## Discussion

The novel findings of this study are 4-fold; 1) AdipoR1 is present in human peripheral resistance arterioles during both health and disease, 2) the adiponectin-mediated restoration of NO-mediated FID in microvessels from individuals with CAD is dependent on AdipoR1, 3) AdipoR1 expression is necessary to maintain NO as the primary mediator of FID during health whereas loss of AdipoR1 does not impair the ability of an arteriole to dilate to flow during disease, and 4) osmotin, a plant-derived protein whose receptor in plants shares homology with AdipoR1, may potentially serve as an adiponectin receptor activator to improve microvascular endothelial health. Microvascular endothelial dysfunction is considered a precursor to ischemic heart disease and potentially heart failure with preserved ejection fraction (HFpEF) (Paulus and Tschöpe, 2013; Sabbatini and Kararigas, 2020). Strategies which promote a healthy microvascular endothelium therefore may prevent or reverse course of disease. This is the first study to highlight the importance of adiponectin receptors, specifically AdipoR1, in restoration and maintenance of a quiescent microvascular endothelium.

### Adiponectin and Vascular Health

Adiponectin is a protein released from adipocytes that exists in three oligomeric forms (high, medium, and low molecular weight) as well as a globular arrangement. The vast majority of studies on adiponectin focus on its role as a regulator of metabolism and energy homeostasis. Hypoadiponectinemia is observed in individuals who are obese, diabetic, and suffering from cardiovascular disease (Pischon et al., 2004). These observations, and the fact that adiponectin has both anti-inflammatory and anti-atherosclerotic effects on arteries (Matsuda et al., 2002; Yamauchi et al., 2003), add strength to the concept that it promotes vascular health. Despite the strong association between low plasma adiponectin and disease, few studies have investigated how the compound affects vascular function *in vivo.* Torigoe et al. demonstrated that reduced HMW adiponectin serves as an independent risk factor for impaired brachial artery flow-mediated dilation (FMD) therefore plasma levels may predict endothelial dysfunction prior to onset of disease (Torigoe et al., 2007), however the findings of this study are limited as it only included healthy men.

Even less is known about the effects of adiponectin on the microvasculature, however we have shown that chronic exposure to either globular adiponectin or AdipoRON, a nonselective adiponectin receptor agonist, restores NO-mediated FID in arterioles from patients diagnosed with CAD (Schulz et al., 2019). The current study confirms that activation of AdipoR1 is necessary to restore NO as the primary mediator of FID as decreased expression of the receptor abolished the effect. Interestingly, AdipoR1 is expressed in arterioles from both healthy individuals as well as those with CAD. Therefore AdipoR1 may be a potential target to improve microvascular health in times of disease.

### Adiponectin and Nitric Oxide Signaling

Two receptors (AdipoR1/R2) have been identified in endothelial cells, however the expression of AdipoR1 is approximately 5-fold higher compared to AdipoR2 (Addabbo et al., 2011). Direct administration of adiponectin triggers the formation of NO in endothelial cells primarily *via* activation of AMPK, PPAR*α*, and PI3k/Akt pathways (da Silva Rosa et al., 2021; Cohen et al., 2022). Upon adiponectin binding to AdipoR1/2, an adapter protein known as APPL1 is necessary for these downstream events and subsequent phosphorylation of endothelial nitric oxide synthase (eNOS) at Ser1177 (Dimmeler et al., 1999). Additionally, adiponectin also stimulates Hsp90 binding to eNOS (Xi et al., 2005) and increases bioavailability of tetrahydrobiopterin (Margaritis et al., 2013), both of which promote NO formation. It was more recently discovered that adiponectin receptors themselves have the intrinsic ability to hydrolyze lipids, specifically sphingolipids including ceramide (Holland et al., 2011). When elevated in plasma this stress lipid serves as an independent risk factor for MACE (Havulinna et al., 2016) and is a known trigger of human microvascular endothelial dysfunction *in vitro* ^11^ *.* Intracellular ceramide concentrations increase in the absence of adiponectin receptors and while the receptors have basal ceramidase activity, the breakdown of ceramide to sphingosine increases dramatically (∼20-fold) in the presence of adiponectin (Vasiliauskaité-Brooks et al., 2017). Here we show that not only activation of AdipoR1 is necessary to restore NO-mediated FID, but decreased expression of AdipoR1 alone is sufficient to promote endothelial dysfunction. Vasodilation to flow was maintained and remained dependent on H_2_O_2_ in vessels from subjects with CAD suggesting that loss of AdipoR1 does not alter the ability to dilate nor the mechanism of dilation during disease. It is possible that basal ceramidase activity in AdipoR1, the most abundant adiponectin receptor within the endothelium (Addabbo et al., 2011), is critical in preventing ceramide accumulation and the transition to H_2_O_2_-dependent FID.

### AdipoR1 as a Therapeutic Target

Although they do not specifically target adiponectin receptors, medications to treat diabetes may also increase plasma adiponectin. These include metformin as well as thiazolidinediones which are known to increase the HMW form that correlates with cardiovascular disease (Miyazaki et al., 2004; Su et al., 2016). These diabetic medications have proven beneficial effects on the vasculature. For instance, vascular endothelial function, as assessed by acetylcholine-induced dilation, was shown to improve in mesenteric arteries from insulin-resistant rats (Katakam et al., 2000) as well as obese, non-diabetic rats treated with metformin (Lobato et al., 2012). It remains unknown whether the increase in adiponectin is responsible for the beneficial vascular effects observed with metformin and other diabetic medications.

Considerable effort has been directed towards the development of adiponectin receptor agonists as they may offer a treatment strategy for metabolic syndrome. AdipoRON, a nonselective, small molecule adiponectin receptor agonist has emerged as a promising agent as it orally active, and through activation of AdipoR1/2, activates AMPK to reduce fasting glucose in obese mice (Okada-Iwabu et al., 2013) similar to metformin, and even prolong their lifespan (Okada-Iwabu et al., 2013). As with exogenous adiponectin, AdipoRON is also capable of restoring NO-mediated FID in vessels from diseased individuals (Schulz et al., 2019). In addition to small molecule agonists it has been suggested that osmotin, a member of the plant pathogenesis-related (PR) protein family, is capable of activating adiponectin receptors (Narasimhan et al., 2005). Exogneous osmotin (0.1–0.3 μM) has been shown to protect against increases in inflammatory cytokines and improve cell viability in an AdipoR1-dependent manner (Liu et al., 2017). Osmotin itself does not share sequence homology with adiponectin however they do both contain a *β*-barrel (Min et al., 2004) and the receptors they bind to (PHO36 and AdipoR for osmotin and adiponectin, respectively) share similar sequences. This stress protein is critical for plant survival and is found in almost all plants but is abundant in tomatoes, peppers, and potatoes (Anil Kumar et al., 2015). Osmotin suppresses inflammatory signaling, vascular cell adhesion molecule-1, E-selectin, and significantly decreases atherosclerotic plaque accumulation in ApoE^−/−^ following chronic infusion (Takahashi et al., 2018). Positive effects have also been confirmed *in vivo* as osmotin through activation of the AdipoR1/2, APPL1, AMPK pathway, reduced blood glucose levels, decreased insulin resistance, and increased fatty acid oxidation and mitochondrial function (Ahmad et al., 2019). Like adiponectin osmotin can also trigger activation of the PI3k/Akt pathway to promote eNOS phosphorylation (Liu et al., 2017).

**FIGURE 5:**
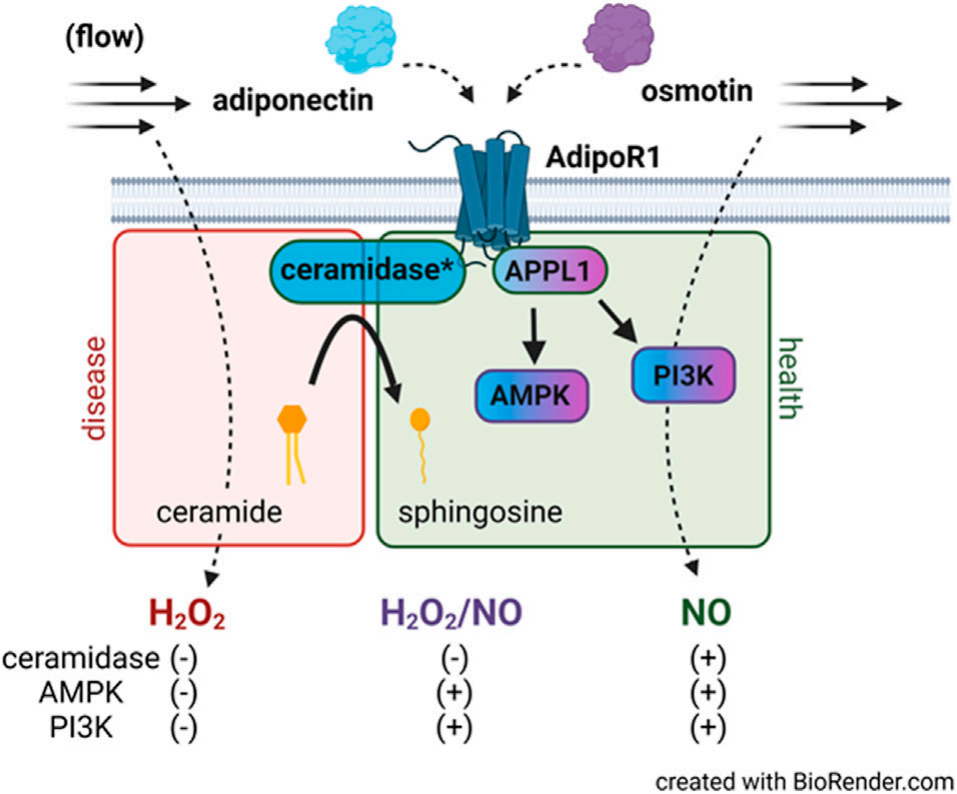
AdipoR1 signaling in the microvascular endothelium. Activation of AdipoR1 by adiponectin (blue) can activate multiple downstream pathways including AMPK and PI3K *via* the adapter protein APPL1. Adiponectin receptors have intrinsic ceramidase activity that allows for hydrolysis of ceramide to sphingosine. Exogenous adiponectin promotes NO-mediated FID in vessels from patients with CAD while accumulation of ceramide in healthy vessels promotes formation of H_2_O_2_ during flow. Osmotin (purple) like adiponectin activates AdipoR1, APPL1, AMPK, and PI3K however dilation to both NO and H_2_O_2_ is observed in arterioles from subjects with disease. Whether osmotin is capable of triggering the ceramidase activity in AdipoR1 remains unknown. *ceramidase activity is within the adiponectin receptor.

Here we show that following incubation with osmotin (16 h, 0.3 μM), human arterioles from CAD patients were able to dilate to both NO and H_2_O_2_ in response to flow. It was only when both L-Name and PEG-catalase were administered simultaneously that a decrease in FID was observed. This plasticity has also been observed in arterioles treated with both adiponectin and ceranib-1, an inhibitor of ceramidase that promotes ceramide accumulation. Although osmotin has proven beneficial effects and like adiponectin, activates the AdipoR-PI3k/Akt-eNOS pathway, it unknown whether osmotin is capable of inducing ceramidase activity within adiponectin receptors. It is possible that activation of the PI3k/Akt-eNOS pathway is allowing for production of NO during flow however when inhibited, the increased ceramide allows for a compensatory pathway in which H_2_O_2_ can be produced to elicit dilation (Figure 5). Although the dose used in this study (0.3 μM) has produced protective effects in cultured endothelial cells, it could be that a higher concentration is needed to achieve complete restoration of NO-mediated FID. Although other studies have shown that siRNA can be used in human vessels without causing damage (Kadlec et al., 2017), it is also plausible that the transfection reagent induces endothelial dysfunction and the effect of osmotin at this concentration produces a more modest effect than adiponectin. Future studies are needed to determine whether osmotin can trigger ceramidase activity in adiponectin receptors and effectively reduce ceramide levels to improve (micro)vascular function.

## Conclusion

The data presented here highlights the importance of AdipoR1 in promoting NO-mediated signaling during flow within the human microvasculature. Endothelial dysfunction within the microvascular network is increasingly recognized as the driver of future large artery disease and cardiac events. Although vasodilation to flow can occur *via* either NO or H_2_O_2_, the effects that each compound has on the surrounding parenchyma is drastically different with the preferred mediator being the anti-inflammatory and anti-atherosclerotic NO. Interventions to treat endothelial dysfunction and restore NO as the primary substance formed during shear may offer an effective treatment strategy to prevent future heart disease. AdipoR1 receptors are present in human arterioles during health and disease and their presence appears critical to maintain NO-mediated FID. AdipoR1 activation can occur through administration of small molecule agonists (AdipoRON) and possibly plant-derived proteins such as osmotin. NO signaling during flow may be promoted by osmotin results in a dual mediator phenotype that may offer plasticity in FID signaling during times of decreased NO bioavailability.

## Data Availability

The original contributions presented in the study are included in the article/Supplementary Material, further inquiries can be directed to the corresponding author.

## Abbreviations

AdipoR1, adiponectin receptor 1; CAD, coronary artery disease; cPTIO, 2-4-carboxyphenyl-4,4,5,5-tetramethylimidazoline-1-oxyl-3-oxide; DM, diabetes mellitus; FID, flow-induced dilation; H_2_O_2_, hydrogen peroxide; L-NAME, N^ω^-nitro-ʟ-arginine; MACE, major adverse cardiac events; NO, nitric oxide; nonCAD, non-coronary artery disease; PEG-Catalase, polyethylene glycol-catalase; ROS, reactive oxygen species.
